# Enteropathy-associated T-cell lymphoma presenting with gastrointestinal tract symptoms: A report of two cases and review of diagnostic challenges and clinicopathological correlation

**DOI:** 10.3892/ol.2014.2105

**Published:** 2014-04-29

**Authors:** GUOHUI JIAO, ZHONGQING ZHENG, KUI JIANG, JIE ZHANG, BANGMAO WANG

**Affiliations:** Department of Gastroenterology, Tianjin Medical University General Hospital, Tianjin 300052, P.R. China

**Keywords:** lymphoma, Crohn’s disease, enteropathy, endoscopy

## Abstract

The gastrointestinal tract is the most common location for primary extranodal non-Hodgkin lymphoma (NHL) with cases less commonly found in the intestine. The majority of primary intestinal B-cell lymphomas are exophytic, whereas enteropathy-associated T-cell lymphomas present predominantly as thickened plaques, ulcers or strictures. Crohn’s disease (CD) is a chronic inflammatory disease of the intestines with fissures and ulcers, which is difficult for clinicians to diagnose based on endoscopic observations alone. Malignant lymphoma must be considered when clinically diagnosed CD is refractory to medication or when its clinical course becomes aggressive. The current study presents a rare case of primary colon T-cell lymphoma in a 16-year-old male with poor prognosis, as well as a case of gastrointestinal lymphoma occurring in the duodenum and colon in a 62-year-old male with a 10-year history of NHL. It was difficult to determine the diagnosis by a single endoscopic biopsy as the majority of biopsy specimens revealed mixed inflammation within which the lymphoma cells were difficult to identify. The present study indicated that it is important to recognize ulcerative or stenotic lymphoma and to differentiate it from CD as it exhibits a much more aggressive clinical behavior. The correct diagnosis may be confirmed by careful histopathological study and ancillary examination.

## Introduction

Extranodal non-Hodgkin’s lymphomas (NHLs) are lymphomas that arise from tissues other than lymph nodes and exhibit a higher incidence in developing countries. The gastrointestinal tract (GIT) is the most frequent primary extranodal localization of NHL. Primary gastrointestinal lymphoma accounts for 4–20% of NHL cases and 30–40% of all extranodal sites, which are predominantly of B-cell origin. Furthermore, the intestine accounts for one-third of all GIT sites involved during the clinical course (1).

The majority of NHL patients are diagnosed with the complaint of diarrhea, or occur secondary to enteropathy. In contrast to B-cell lymphoma, enteropathy-associated T-cell lymphoma (ETCL) is a highly aggressive T-cell lymphoma with a poor prognosis (2) and due to non-specific clinical and endoscopic observations, early diagnosis and appropriate treatment may be delayed (3).

The current study presents a case of intestinal T-cell lymphoma, as well as a case of secondary B-cell lymphoma complicated by intestinal Crohn’s disease (CD), to investigate the clinicopathological features and immunophenotype of lymphoma and its differentiation with CD. The study was approved by the ethics committee of the Tianjin Medical University General Hospital. Patients provided written informed consent.

## Case report

### Case one

In December 2011, a 16-year-old male with complaints of abdominal pain and bloody diarrhea for one day was admitted to the Tianjin General Hospital (Tianjin, China). The patient had a history of low-grade fever and intermittent abdominal pain without diarrhea of one month. No enlarged lymph nodes or hepatomegaly and splenomegaly were identified by physical examination and color Doppler ultrasonography (Prosound SSD-α5; Aloka Co., Ltd., Tokyo, Japan) revealed no lymph node enlargement in the chest or pelvic cavity. In addition, an abdominal computed tomography (CT) scan revealed thickening of the wall of the initial segment of the ascending colon without evidence of intraabdominal lymph node enlargement. The laboratory tests revealed moderate anemia with a hemoglobin concentration of 60–80 g/l, a normal erythrocyte sedimentation rate and a C-reactive protein concentration of 2.3 mg/l. The acid-fast stain of the colonic biopsies was negative for acid-fast bacilli and the purified protein derivative test was negative with antibody positive results. The patient was also negative for human immunodeficiency virus (HIV), cytomegalovirus (CMV) and hepatic virus infection. However, the patient was EB-IgG (+) with Epstein-Barr virus (EBV) negative DNA.

**Figure 1:**
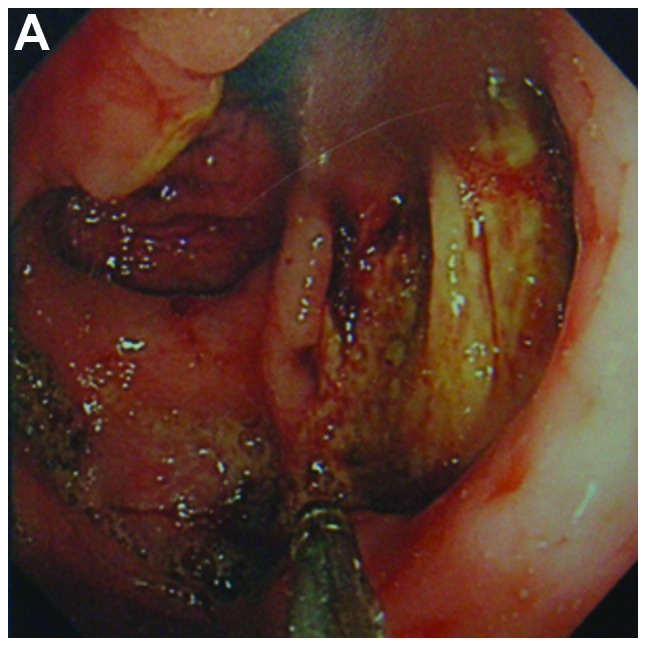

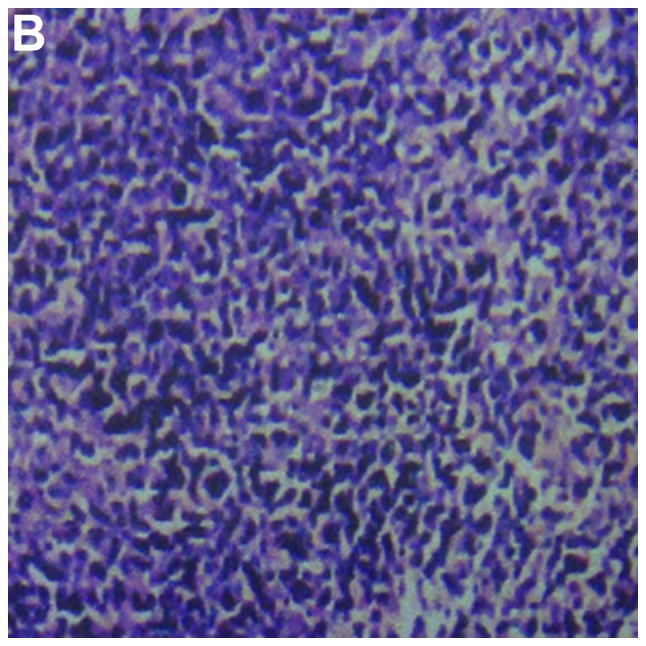
(A) Gastroendoscopy revealed a 2×3.5-cm ulcer in the ileocecum and multifocal irregular ulcers distributed circumferentially or transversely in the distal intestine. Biopsy was performed in a different location. (B) Hematoxylin-eosin staining revealed suppurative colitis accompanied by focal necrosis. Atypical granuloma and diffuse proliferation of large-sized atypical lymphoid cells were observed in the section with an inflammatory background.

The gastroendoscopy identified chronic gastritis and total colonoscopy on admission revealed a 2×3.5-cm ulcer in the ileocecum with multifocal irregular ulcers distributed circumferentially or transversely in the distal intestine (Fig. 1A). Furthermore, the histological observations of the specimens revealed active chronic colitis characterized by lymphocyte infiltration. Based on these results, the patient was diagnosed with CD and mesalazine (1 g) was orally administered three times daily as a maintenance therapy, which improved the abdominal pain. However, the patient continued to exhibit a fever, which fluctuated between 37 and 40°C, as well as bloody diarrhea and the occasional presentation of bradycardia, which suggested the possibility of malignant lesions. Four days later, the colonoscopy was repeated and revealed an increased number of irregular ulcers between the ileocecum and the descending colon than previously observed.

The multifocal biopsy specimens revealed extensive suppurative colitis accompanied by focal necrosis. Atypical granuloma and diffuse proliferation of large-sized atypical lymphoid cells were also identified in the section. In addition, mixed inflammatory infiltrates containing small lymphocytes, plasmacytes, neutrophils, eosinophils and histiocytes were identified in the histological background. Furthermore, immunohistochemistry confirmed these atypical cells to be positive for CD3 and negative for CD20 (Fig. 1B), implicating T-cell lymphoma, however, a bone marrow smear revealed no infiltrate of abnormal cells.

Thus, lymphoma was suspected and mesalazine was replaced by prednisone (40 mg daily). However, the patient’s symptoms persisted and liver function continued to deteriorate with severe, ongoing hematochezia. The patient succumbed to hemorrhagic shock two weeks following admission.

### Case two

In January 2011, a 62-year-old male was admitted to the Tianjin General Hospital (Tianjin, China) due to haematemesis and melena with abdominal pain of one month. The patient had a history of NHL of >10 years, which was treated by hematopoietic stem cell transplantation (HST) followed by radiotherapy, which had achieved a sustained response. The patient had no history of diarrhea or malabsorption. The laboratory tests revealed mild hypoalbuminemia and moderate anemia, however, the patient’s liver and renal function test results were within the normal limits. In addition, no evidence of HIV, CMV, EBV or human T-lymphotropic virus infection was identified and among the serum tumor markers, only ferritin was elevated to >2,000 ng/dl.

The patient had intermittent vomiting and anorexia and contrast-enhanced CT of the abdomen and pelvis revealed pneumatosis in the intestine and colon with segmental thickening of the duodenum and ascending colon. In addition, the gastroduodenoscopy revealed a huge duodenal ulcer with stricture, which exhibited no malignant cells or characteristics of enteropathy, as confirmed by biopsy. Masalazine (ASA; 1 g) treatment was resumed, which allowed diarrhea control but no obstruction recovery.

Following supportive treatment, the patient continued to have a fever of >38°C, however, the symptoms improved following nasojejunal nutrition tube implantation. The enteroscopy was repeated and the multifocal biopsy specimens revealed dense lymphocyte and plasma cell infiltration in the mucosal layer. Additionally, immunohistochemistry and flow cytometry revealed that the intraepithelial lymphocytes were predominantly CD20^+^ and CD79a^+^, but negative for CD3, CD117, CD34 and CK20. The patient was subsequently diagnosed with B-cell lymphoma and thus received seven cycles of chemotherapy based on the R-CHOP modality (rituximab, cyclophosphamide, doxyorubicin, vincristine, prednisolone) determined by the Institute of Hematology and Hospital of Blood Diseases, Chinese Academy of Medical Sciences (Tianjin, China).

A follow-up CT scan six months after diagnosis revealed remission of the bowel wall, however, swollen lymph nodes in the mesentery and para-aorta were observed. The gastroduodenoscopy identified ulcers and stricture as previously observed and intestinal biopsies revealed lymphadenosis in the lamina propria with CD3-positive cells and Bcl-2 expression. The patient completed the seven cycles of chemotherapy and achieved a complete response and remains disease free at present. The patient continues to be monitored for disease recurrence during the therapeutic process.

## Discussion

The two cases presented in the current study highlight the importance of correctly diagnosing NHLs of the GIT in patients with different prognoses. The limited pathological data obtained from the biopsies presented a challenge, however, the clinical course provided more information.

In Asia, T-cell lymphoma predominantly arises in young males with a poor prognosis (4). The intestinal T-cell lymphoma is endoscopically characterized by focally, multifocally or diffusely distributed polymorphic irregular ulcers, which most frequently involve the ileocecum and colon. Its location in the intestine is associated with enteropathy and develops from the intraepithelial T-lymphocytes of the intestine (5). ETCL is an aggressive malignancy and if left untreated, leads invariably to mortality due to multifocal intestinal perforation caused by refractory malignant ulcers. The first case presented in the current study progressively deteriorated clinically with a poor nutritional and immunological condition, which prevented the use of adequate and opportune treatment. However, it is possible to speculate late diagnosis and poor performance status at the time of presentation (6). Bulky lesions, stage, histology, immunophenotype, B-cell symptoms and lactate dehydrogenase have all been recognized as the main prognostic indicators and thus, the adverse prognostic features exhibited by this patient caused the colonoscopy to be repeated and a final diagnosis to be reached.

Notably, the patient suffered bradycardia associated with EBV infection and according to previous studies, T-cell lymphoma is markedly associated with EBV infection, with the infectious frequencies ranging between 76.0 and 97.6% in certain Asian populations (7). It has also been recognized that EBV may exhibit a ‘start-up and promotion’ function in the pathogenesis of natural killer/T-cell lymphoma rather than a secondary event. EBV is often implicated in the pathogenesis of lymphoma in primary immunodeficiencies, although, the presence of virus is not always detected, as observed in case one of the present study (8). Notably, Dayharsh *et al* (9) revealed that irritable bowel disease patients who were treated with thiopurines and subsequently developed lymphoma were more likely to exhibit tumors positive for EBV.

In Asian countries, including China, intestinal T-cell lymphoma and tuberculosis are prevalent and the incidence of CD has also increased over recent decades. Askling *et al* (10) suggested that patients with CD carry a risk of malignant lymphoma, which is 30% higher than that of the general population. However, differential diagnosis based on the clinical, endoscopic and histological presentations has become challenging. Although ulcers in lymphoma mimic those of CD, they are transverse in direction as opposed to the usual longitudinal ulcers observed in CD (11). Clinically, intestinal T-cell lymphoma has an aggressive course with poor prognosis, whereas CD exhibits a remitting/relapsing or persistent course and usually remains for life. The administration of immunosuppressive treatments due to a misdiagnosis of CD may delay the diagnosis of malignant lymphoma in such patients. In the current study, it was difficult to reach the lesion in the intestine and obtain the specimen by forceps, however, the histological appearance was atypical. This made it difficult to determine a diagnosis. Thus, close endoscopic surveillance and repetitive inspections may be of great importance in intestinal ulcerative lesions.

In long standing cases, such as the second case presented in the current study, a primary deficiency in B-cell function may be speculated. The majority of primary intestinal lymphomas are of B-cell lineage and predominantly high-grade tumors. In addition, the majority of patients exhibit B symptoms, including weight loss and poor performance status and present with advanced stage lymphoma (12). This may imply that it is a defect in the immunosurveillance, in the detection and destruction of neoplastic cells, which gives rise to the secondary lymphoma. Case two presented in the current study was diagnosed with duodenal B-cell lymphoma 10 years following the initial clinical manifestation of NHL with progressive fever and anemia, as well as clinical symptoms similar to those of inflammatory bowel disease.

This case was also atypical, as the initial colonoscopy and abdominal imaging were negative for any malignancy or lesions. The patient remained disease-free following HST for a relatively long time period. However, within several weeks, the lymphoma had progressed with stricture extension in the duodenum. In the first endoscopy performed on admission, although the mucosa showed diffuse infiltration of lymphocytes with lymphoepithelial lesions on endoscopic biopsy, immunohistochemical stains for B- and T-cell markers were negative. However, the diagnosis of lymphoma was suspected based on the therapeutic presentation of the patient on 5-ASA medication. The second endoscopy was performed to achieve a deeper view of the pathogenesis. The chemotherapy administered for the lymphoma achieved an improved response and the clinical symptoms subsided following the seven cycles of chemotherapy. However, biopsy of the colon mucosa revealed mild proliferation of small lymphocytes.

The aforementioned intestinal diffuse B-cell lymphoma with stricture and causing abdominal pain mimicked the CD pathogenesis. This lymphoma and is likely to be secondary to the immunodeficiency which developed due to a pre-existing lymphoma diagnosed >10 years earlier. Such an uncommon association must be considered when diagnosing and treating patients (13). Previous studies have suggested that to differentiate lymphoma clinically simulating CD, gene rearrangement analysis may aid in the diagnosis of malignant lymphoma when traditional histological and immunohistochemical studies fail to provide a definitive diagnosis (14).

In conclusion, intestinal T/B-cell lymphomas may masquerade as CD or infectious disorders. However, there may be a potential causal association between CD and malignant lymphomas. To address these questions, revealing the nature of lymphomagenesis in association with inflammation is of great significance (15). The current study presents two cases with different outcomes and the manner in which clinicians can acknowledge the crucial point for early diagnosis or optimal intervention must be investigated. To the best of our knowledge, the initial clinically suspected malignant lymphoma with negative histological evidence must not be ignored completely. When the clinical condition appears medically unresponsive, repeated endoscopy with deep biopsies is recommended. Furthermore, immunophenotypic studies and gene analysis may aid clinical decisions, as well as for surgery.
